# A labeled dataset for osteoporosis screening based on electromagnetic attenuation

**DOI:** 10.3389/fdgth.2025.1538477

**Published:** 2025-05-22

**Authors:** Dionísio D. A. Carvalho, Mikael M. R. Costa da Silva, Gabriela A. Albuquerque, Agnaldo S. Cruz, Felipe Fernandes, Ingridy M. P. Barbalho, João Paulo Q. Santos, Antonio H. F. Morais, Karilany D. Coutinho, Antonio L. P. S. Campos, Paulo Gil, César Teixeira, Jorge Henriques, Guilherme M. Machado, Ricardo A. M. Valentim

**Affiliations:** ^1^ Laboratory for Technological Innovation in Health, Federal University of Rio Grande do Norte, Natal, RN, Brazil; ^2^ The Health Sciences Research Unit, Nursing, Nursing School of Coimbra, Coimbra, Portugal; ^3^ Advanced Nucleus for Technological Innovation (NAVI), Federal Institute of Rio Grande do Norte (IFRN), Natal, RN, Brazil; ^4^ Electrical and Computer Engineering Graduate Program, Federal University of Rio Grande do Norte, Natal, RN, Brazil; ^5^ Centre of Technology and Systems (CTS-UNINOVA), NOVA School of Science and Technology, Campus de Caparica, Caparica, Portugal; ^6^ University of Coimbra, Centre for Informatics and Systems of the University of Coimbra, Coimbra, Portugal; ^7^ Research Department, ECE-Engineering School, Paris, France

**Keywords:** public health, machine learning, artificial intelligence, screening, electromagnetic waves

## 1 Introduction

Osteoporosis is an asymptomatic disease characterized by bone microarchitecture deterioration due to multiple risk factors, such as low bone mineral density (BMD), nutritional deficiency, a sedentary lifestyle, alcohol use, smoking, genetic factors, and the use of medications such as glucocorticoids, for example (1–4). The worldwide prevalence of osteoporosis is estimated to be 23.1% in women and 11.7% in men (5). The economic burden related to osteoporosis fractures is significant, costing approximately $17.9 billion a year in the US alone (6).

Studies show that the early and systematic identification of people with clinical indicators of osteoporosis, combined with primary care, appropriate interventions, and the use of therapeutic drugs has reduced the risk of fractures (7, 8). Clark and colleagues (9) demonstrated in a randomized clinical trial that primary healthcare screening tools increased osteoporosis drug prescriptions by 124% and reduced the incidence of fractures in the observed group. Their results support the premise that improving preventive screening methods is essential for identifying individuals at high risk of osteoporotic fractures. Therefore, the development of low-cost digital health technologies or solutions for rapid diagnosis in primary health care is essential.

Early screening for osteoporosis is a challenging problem because the most appropriate diagnostic method, based on dual-energy X-ray absorptiometry (DXA) to measure the number of grams of mineral per square centimeter (g/${\mathrm{cm}}^{2}$) (from the lumbar spine, total femur, femoral neck or middle third of the radius) (10, 11), is highly expensive. Furthermore, as this method uses ionizing radiation technology and requires large, specialized equipment, these devices are generally concentrated in major healthcare centers. Given Brazil’s social inequality and geographical challenges, access to such services is often bureaucratic, slow, and, in some cases, unfeasible.

To overcome these challenges Brazilian researchers have developed a low-cost, portable device for osteoporosis screening called OSSEUS (12, 13). The device combines techniques for measuring the attenuation of electromagnetic waves passing through bone tissue, extracting patient features(risk factors), and recognizing patterns to help classify osteoporosis and provide decision support for healthcare professionals (14). A primary health care screening study using OSSEUS with a group of 505 people referred for a DXA scan found that 78.2% could start preventive care. In addition, the study also showed that 110 people (21.8%) were healthy (concerning this pathology) and did not need to be referred to specialized health centers to get a DXA screening (15).

Recognizing the importance of osteoporosis screening in primary health care and early care, such as treatment to reduce the incidence of fractures—especially in people identified by OSSEUS as having risk factors for the disease—, this study aims to provide a database of 669 people available to support future research, especially in the field of artificial intelligence and machine learning, and contribute to the development of digital health solutions in response to osteoporosis. The dataset is available at: https://doi.org/10.5281/zenodo.14259374.

## 2 Materials and methods

### 2.1 Study design and participants

This study consists of a data report conducted and guided by a descriptive analysis of osteoporosis and innovative technologies enabling early disease detection. The dataset comprises 669 people (575 females, 94 males), 156 from the control group, aged between 20–85 years (median age: 55; mean age: 54.5 ± 13.2), and 513 with low BMD, aged between 18–101 years (median age: 66; mean age: 65.3 ± 11.2). Table 1 shows the demographic, anthropometric, risk factor, OSSEUS, and DXA features of the population in the dataset. All participants in the dataset were volunteers who met the eligibility criteria. Participation was limited to individuals of both sexes, aged 18 or older, with a medical indication (prescription/request) for DXA. In addition to these criteria, participants were required to have intact middle finger phalanges to meet specific prerequisites for OSSEUS.

**Table 1:** General characteristics of dataset.

Features	Male (n=94)		Female (n=575)	
	*n*	(14,05%)	*n*	(85,95%)
Electronic health record				
Age (mean ± SD, min-max)	61 ± 15, 18–85		63 ± 12, 19–101	
Height (mean ± SD, cm)	163,1 ±12,9		151,8 ± 10,2	
Weight (mean ± SD, kg)	74,1 ± 15,1		67,0 ± 16,6	
Ethnicity (black)	12	12,7%	18	3,1%
Ethnicity (brown)	32	34,0%	170	29,5%
Ethnicity (white)	50	53,1%	387	67,3%
Alcohol	29	30,8%	84	14,6%
Smoking	40	42,5%	211	36,6%
Activity	42	44,6%	173	30,0%
Milk	65	69,1%	455	79,1%
Calcium	20	21,2%	217	37,7%
Vitamin D	50	53,1%	278	48,3%
Fall	22	23,4%	110	19,1%
Parents osteoporosis	23	24,4%	195	33,9%
Parents curved	8	8,5%	38	6,6%
Corticosteroids	23	24,4%	132	22,9%
Arthritis	1	1,0%	59	10,2%
Diseases	41	43,6%	260	45,2%
Menopause	0	0%	519	90,2%
Target (normal)	40	42,5%	116	20,1%
Target (LBMD)	35	37,2%	274	47,6%
Target (osteoporosis)	19	20,2%	185	32,1%
OSSEUS				
Medial length (mean ± SD, cm)	28,7 ± 2,2		27,1 ± 2,2	
Medial height (mean ± SD, cm)	14,7 ± 1,5		12,5 ± 1,1	
Medial width (mean ± SD, cm)	16,2 ± 1,5		14,1 ± 1,2	
Calibration (mean ± SD, mV)	1667,1 ± 51,5		1685,8 ± 42,6	
Attenuation (mean ± SD, mV)	1255,6 ± 109,2		1304,2 ± 110,8	
DXA				
Deviation (mean ± SD)	−1,4 ± 1,5		−2,0 ± 1,1	

#### 2.1.1 Ethical approval

The experimental protocol was approved by the Research Ethics Committee (CEP) of the Federal University of Rio Grande do Norte, Natal, Brazil, through a letter, under CAAE No. 39675020.0.0000.5292/2020, as well as by the Federal Institute of Rio Grande do Norte, Natal-RN, Brazil, an official letter; under CAAE No. 75015123.9.0000.0225/2023, and following the Helsinki Accords (as amended in 2004).

### 2.2 Procedures

#### 2.2.1 Data collection

The data from the 669 people was collected at the Onofre Lopes University Hospital (HUOL) at the Federal University of Rio Grande do Norte (UFRN), between July 2021 and November 2023. The sampling procedure tookan average of 20 min per person and consisted of three stages: (i) completion of anamnesis in the Electronic Health Record (EHR) for collection of clinical data and identification of risk factors for metabolic bone diseases; (ii) performing anthropometry with a pachymeter on the medial phalanx of the middle finger of the non-dominant hand, followed by measurement of radiofrequency signals using OSSEUS; (iii) acquisition of standard deviations calculated by DXA (GE Lunar DPX Pro) at the sites the scan was performed (10).

**Figure 1:**
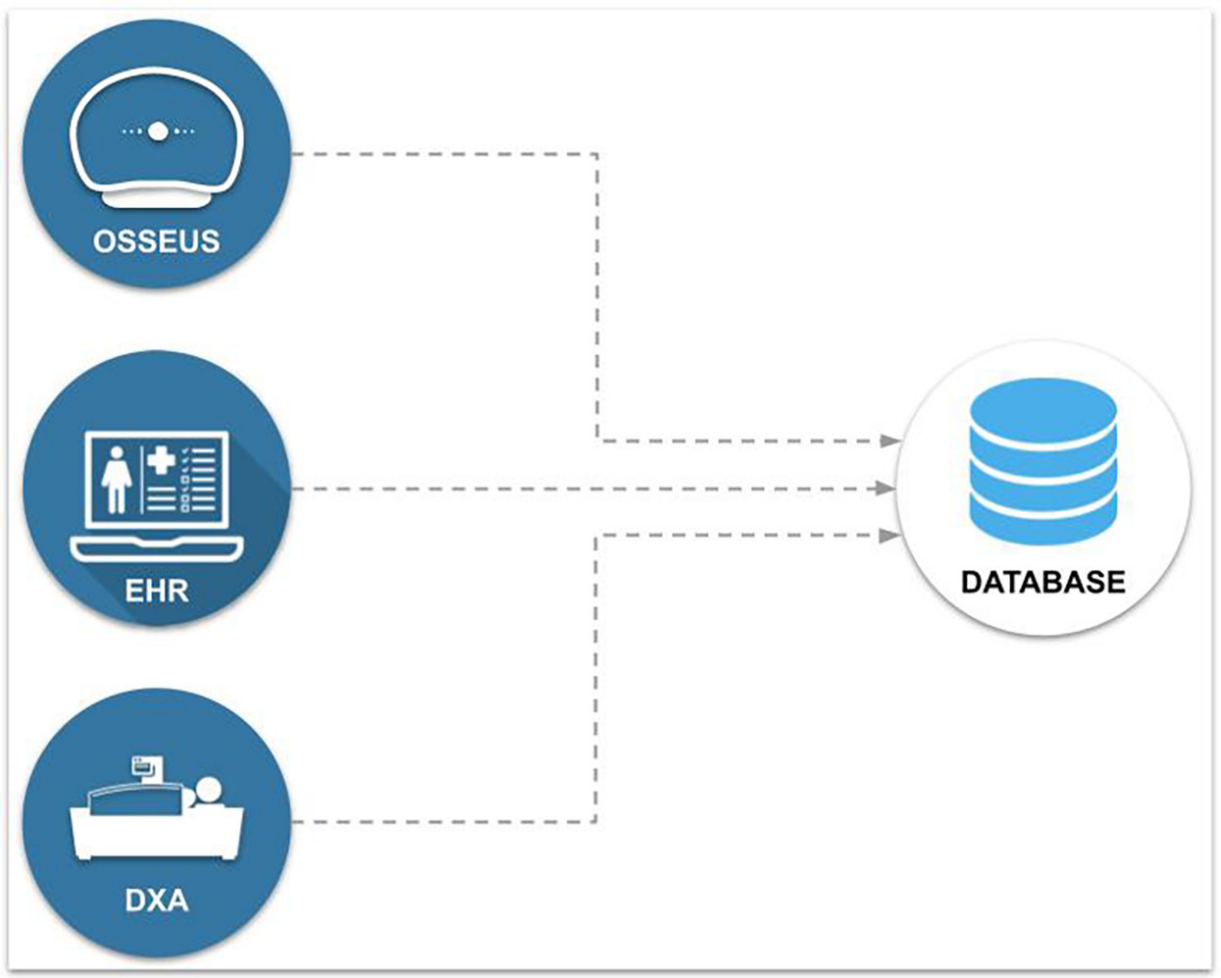
Architecture for integrating data into a database.

Figure 1 illustrates the architecture designed for integrating the three distinct data sources into a centralized database. Each person in those data sources was identified with a unique hash code, ensuring data integrity when building the consolidated database. The database includes 29 features, which have been duly anonymized. A subset of features from a subgroup of individuals in the database was used as predictor variables in a preliminary machine learning study to predict referrals for bone densitometry tests (15).

#### 2.2.2 Data preprocessing

The data were inspected for instances with missing values or values outside the normal range (outliers). Instances with missing values for attributes related to age and the DXA scans were removed. Next, the data generated by the OSSEUS device was inspected and the instances with attenuation values exceeding those obtained during calibration were removed—since results falling outside the calibrated range may indicate measurement errors or malfunctioning of the device, compromising the validity and quality of the readings.

A new attribute, named’ worst deviation’, was defined to store the lowest value among the four possible sites DXA can be performed. This attribute, along with age, gender, and menopause attributes, is considered in calculations for a diagnostic definition of metabolic bone diseases, as recommended by the Brazilian Ministry of Health in its Clinical Protocol and Therapeutic Guidelines for Osteoporosis (PCDT) (10). In summary, based on the calculation of clinical variables, menopausal women and individuals aged 50 or older are diagnosed using the T-score deviation. The remaining registries use the Z-score for diagnosis (16). Deviation values below −2.0 in the Z-score or below −1.0 in the T-score are classified as “low BMD” or “osteoporosis” (17).

The dataset also enables feature engineering, i.e., the definition, creation, or aggregation of predictive attributes. For instance, it is possible to calculate the Body Mass Index (BMI) from Electronic Health Record (EHR) data, considering body weight (kg), height (m), and the following equation:

$$\mathrm{BMI}=\frac{\mathrm{weight}}{{\mathrm{height}}^{2}}.$$

In addition, it is possible to conduct investigative research using OSSEUS data, considering the real or percentage differences between the calibration and attenuation attributes of the biomedical device and the influence of obstacles in this context. The obstacle can be determined by estimating the area or volume of the medial phalanx of the middle finger, using the following equations:

$$\mathrm{area}=2\pi r(r+h);\;\mathrm{volume}=\pi r^{2}h,$$

where:

– $d=\frac{\mathrm{medial}\_\mathrm{height}+\mathrm{medial}\_\mathrm{width}}{2}$, determines the diameter of the medial phalanx of the middle finger;
– $r=\frac{d}{2}$, defines the medial phalanx radius of the middle finger;
– h=medial_length, represents the length of the medial phalanx of the middle finger.

## 3 Descriptive analysis

The dataset presents relevant characteristics of a particular region of Brazil, which can significantly contribute to training artificial intelligence algorithms, especially machine learning. The DXA screening reports certified by specialized health professionals indicated that 156 (23.32%) individuals had normal BMD, 309 (46.19%) had low BMD, and 204 (30.49%) had osteoporosis. In the sample, 609 (91.10%) were eligible for analysis based on the T-score and 60 (8.90%) on the Z-score, as recommended by the Clinical Protocol and Therapeutic Guidelines for Osteoporosis (PCDT) (10).

**Figure 2:**
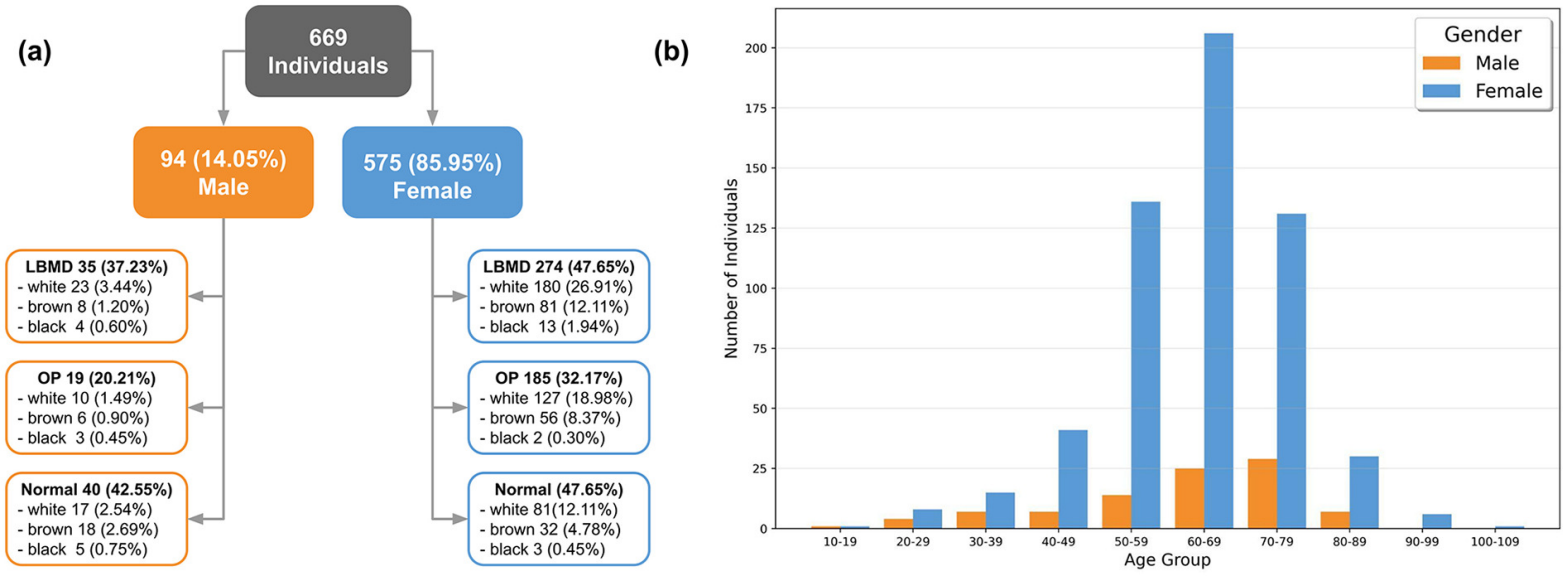
Analysis summary. **(a)** Overview of individuals’ features. **(b)** Age distribution by gender. LBMD, low bone mineral density; OP, osteoporosis.

Moreover, based on gender features, 575 (85.95%) participants were females, and 94 (14.05%) were males. Figure 2a breaks down the most relevant features of both groups, considering the disease-related outcome. Figure 2b displays the age range of the studied group and shows a higher concentration of people in the 50–79 age bracket, totaling 541 (80.87%) individuals.

One of the groups recommended for DXA screening includes women over 65 and men aged 70 or older (10). Such group comprises 312 (46.6%) individuals with an average age of 72.8 years (ranging from 65 to 101 years), including 33 (10.5%) with normal BMD, 149 (47.7%) with low BMD, and 130 (41.6%) with osteoporosis.

Data stratification revealed that 252 people (37.67%) reported smoking, 215 (32.14%) were physically active, and 218 (32.59%) had osteoporotic parents. In addition to these characteristics, the analysis revealed a group of 302 (45.14%) individuals with underlying comorbidities and a second group of 132 (19.73%) who had previously experienced falls. Considering only the female group, which accounts for 575 participants, the data show that 519 (90.26%) reported being menopausal. In the male group, there were no reports of testosterone use. Additional characteristics can also be noted in the dataset, such as milk intake (77.7% reported consuming it) and supplementation with calcium (35.58%) and vitamin D (49.18%).

## Data Availability

The datasets presented in this study can be found in online repositories. The names of the repository/repositories and accession number(s) can be found below: The dataset analyzed for this study can be found in the Zenodo: https://doi.org/10.5281/zenodo.14259374.

## References

[1] Albuquerque G Cruz A Carvalho D Mayrink N Pinheiro B Campos A A method based on non-ionizing microwave radiation for ancillary diagnosis of osteoporosis: a pilot study. Biomed Eng Online. (2022) 21:70. 10.1186/s12938-022-01038-y 36138480 PMC9494783

[2] Compston JE McClung MR Leslie WD. Osteoporosis. Lancet. (2019) 393:364–76. 10.1016/S0140-6736(18)32112-3 30696576

[3] Cruz AS Lins HC Medeiros RVA Filho JMF da Silva SG. Artificial intelligence on the identification of risk groups for osteoporosis, a general review. Biomed Eng Online. (2018) 17:12. 10.1186/s12938-018-0436-1 29378578 PMC5789692

[4] Pouresmaeili F Kamalidehghan B Kamarehei M Goh Y. A comprehensive overview on osteoporosis and its risk factors. Ther Clin Risk Manag. (2018) 14:2029–49. 10.2147/TCRM.S138000 30464484 PMC6225907

[5] Salari N Ghasemi H Mohammadi L Behzadi MH Rabieenia E Shohaimi S The global prevalence of osteoporosis in the world: a comprehensive systematic review and meta-analysis. J Orthop Surg Res. (2021) 16:609. 10.1186/s13018-021-02772-0 34657598 PMC8522202

[6] Clynes MA Harvey NC Curtis EM Fuggle NR Dennison EM Cooper C. The epidemiology of osteoporosis. Br Med Bull. (2020) 133:105–17. 10.1093/bmb/ldaa005 32282039 PMC7115830

[7] Chotiyarnwong P McCloskey EV Harvey NC Lorentzon M Prieto-Alhambra D Abrahamsen B Is it time to consider population screening for fracture risk in postmenopausal women? a position paper from the international osteoporosis foundation epidemiology/quality of life working group. Arch Osteoporos. (2022) 17:87. 10.1007/s11657-022-01117-6 35763133 PMC9239944

[8] Curtis EM Dennison EM Cooper C Harvey NC. Osteoporosis in 2022: care gaps to screening and personalised medicine. Best Pract Res Clin Rheumatol. (2022) 36:101754. 10.1016/j.berh.2022.101754. 35691824 PMC7614114

[9] Clark EM Gould V Morrison L Ades A Dieppe P Tobias JH. Randomized controlled trial of a primary care–based screening program to identify older women with prevalent osteoporotic vertebral fractures: cohort for skeletal health in bristol and avon (coshiba). J Bone Miner Res. (2012) 27:664–71. 10.1002/jbmr.1478 22113935 PMC3378696

[10] Brasil. Protocolo clínico e diretrizes terapêuticas da osteoporose (2023). Ministério da Saúde. Secretaria de Atenção Especializada à Saúde. Secretaria de Ciíncia Tecnologia, Inovação e Complexo da Saúde. Available at: https://www.gov.br/saude/pt-br/assuntos/pcdt/arquivos/2023/portaria-conjunta-no-19-pcdt-osteoporose (Accessed February 29, 2024).

[11] Kanis JA. Assessment of fracture risk and its application to screening for postmenopausal osteoporosis: synopsis of a who report. Osteoporos Int. (1994) 4:368–81. 10.1007/BF01622200 7696835

[12] Cruz AS. Osseus: método baseado em inteligência artificial e ondas eletromagnéticas para o diagnóstico auxiliar de doenças osteometabólicas (Ph.D. thesis). Universidade Federal do Rio Grande do Norte, Centro de Tecnologia, Programa de Pós-Graduação em Engenharia Elétrica e de Computação, Natal, Rio Grande do Norte, Brazil (2018). p. 121f.

[13] Cruz AS da Silva SG de Castro BH. Bone density measurement through electromagnetic waves. In: The 6th 2013 Biomedical Engineering International Conference. (2013). p. 1–5. 10.1109/BMEiCon.2013.6687655

[14] Pinheiro BM Campos ALPS de Carvalho DDA Cruz AS de Medeiros Valentim RA Veras NVR The influence of antenna gain and beamwidth used in osseus in the screening process for osteoporosis. Sci Rep. (2021) 11:19148. 10.1038/s41598-021-98204-4 34580323 PMC8476524

[15] Albuquerque GA Carvalho DDA Cruz AS Santos JPQ Machado GM Gendriz IS Osteoporosis screening using machine learning and electromagnetic waves. Sci Rep. (2023) 13:12865. 10.1038/s41598-023-40104-w 37553424 PMC10409756

[16] Brandão CMA Camargos BM Zerbini CA Plapler PG de Carvalho Mendonça LM Albergaria B-H Scielo – Brasil – posições oficiais 2008 (2009). Posições Oficiais 2008 da Sociedade Brasileira de Densitometria Clínica (SBDens). Available at: https://www.scielo.br/j/abem/a/jMQwSSZVxQmLXqYxt4zjNkv (Accessed April 10, 2024). 10.1590/s0004-27302009000100016 19347193

[17] Netto OS de Oliveira Lima Coutinho L de Souza DC. Análise da nova classificação de laudos de densitometria óssea. Radiol Bras. (2007) 40(1):23–5. 10.1590/S0100-39842007000100007

